# Comparison of the Mechanical Properties and Crack Expansion Mechanism of Different Content and Shapes of Brass-Coated Steel Fiber-Reinforced Ultra-High-Performance Concrete

**DOI:** 10.3390/ma16062257

**Published:** 2023-03-11

**Authors:** Yanli Jiang, Yulong Yan, Tianran Li, Xiuling Cao, Liang Yu, Haiquan Qi

**Affiliations:** 1 Key Laboratory of New Processing Technology for Nonferrous Metals & Materials, Guilin University of Technology, Guilin 541004, China; 2 Guangxi Key Laboratory of Optical and Electronic Materials and Devices, Guilin 541004, China; 3 Guangxi Modern Industry College of Innovative Development in Nonferrous Metal Material, Guilin 541004, China; 4 Hebei Technology Innovation Center for Intelligent Development and Control of Underground Built Environment, Shijiazhuang 050031, China; 5 School of Urban Geology and Engineering, Hebei GEO University, Shijiazhuang 050031, China

**Keywords:** ultra-high performance concrete, brass-coated steel fibers, flexural strength, compressive strength, crack extension mechanism, microstructure

## Abstract

Steel fiber-reinforced ultra-high-performance concrete (UHPC) is becoming an important type of concrete reinforcement. After mixing with the reinforced steel fibers, the UHPC has perfect flex resistance, shear strength, crack resistance, shock resistance, and anti-seepage. In this study, the influence of straight, corrugated, and hooked brass-coated steel fibers (BCSFs) on the microstructure, mechanical properties, and crack expansion mechanism of ultra-high-performance concrete (UHPC) with varying content of 1–6 wt.% under different curing times were investigated. Field emission scanning electron microscopy and energy dispersive X-ray spectrometry were employed to characterize the microstructure of the BCSF-reinforced UHPC mix specimens. X-ray computed tomography was employed to determine the porosity of the BCSF-reinforced UHPC mix specimens. The obtained results indicate the flexural strength and compressive strength of BCSF-reinforced UHPC mix specimens are enhanced, along with increasing the content of BCSFs reinforcement with different shapes (straight, corrugated, and hooked). The embedded BCSFs play a major role in the adhesive property and stress transfer of the BCSFs–UHPC matrix interface. Different from many studies, the flexural strength of mix UHPC with straight BCSFs is higher than those with corrugated and hooked BCSFs. However, the compressive strength of UHPC with corrugated BCSFs is higher than those with straight and hooked BCSFs. The flexural strength of mix UHPC with 6 wt.% straight BCSFs at 28 days reaches the maximum value of 26.2 MPa, and the compressive strength of UHPC with 6 wt.% corrugated BCSFs at 28 days reaches the maximum value of 142.3 MPa. With the increase in straight BCSF content from 1 wt.% to 6 wt.%, the porosity in mix UHPC reduces gradually from 18.4% to 8.3%. The length of average crack spacing is dependent on the straight BCSF content. With the increase in straight BCSF content from 1 wt.% to 6 wt.%, the average crack length reduces gradually from 34.2 mm to 12.1 mm, and the average crack width reduces gradually from 0.78 mm to less than 0.1 mm. During crack extension, part of the energy in the UHPC mixture specimen with the 6 wt.% BCSF content flows into the crack tip region converted into the work dissipated during the bridging process. The crack propagation resistance of the UHPC mixture with straight BCSFs was improved compared with those with corrugated and hooked BCSFs. The bond strength between the BCSFs and UHPC matrix was enhanced by using vibrational mixing, and the brass film coated on the BCSFs contributes to increase the flexural and compressive strength of the UHPC mixture.

## 1. Introduction

Ultra-high-performance concrete (UHPC) is obtained by adding ultra-fine auxiliary cementitious materials and high-efficiency water reducing agents to reduce the water–cement ratio, reduce porosity, and increase compactness under the premise of satisfying working strength [1]. The steel fiber, as one of the popular admixtures of UHPC, can have a significant impact on the properties and failure process of UHPC under dynamic loading [2,3]. Steel fibers can also control the opening of diagonal cracks and enhance aggregate interlocking [3]. Due to the good strength of steel fiber-reinforced UHPC, it is now widely used in the bridge civil engineering, water conservancy, explosion-proof engineering, national defense, and military engineering fields [4].

Yang studied the effects of steel fibers on the dynamic properties and failure process of ultra-high-performance concrete [1]. Xiong studied SFRLAC that was prepared based on ordinary mixing and vibrational mixing, respectively. Wu studied the static and dynamic compressive properties of UHPC with hybrid steel fiber reinforcements [5]. Bae studied the effect of steel fiber content on the structural and electrical properties of UHPC sleepers [6]. Isa developed an affordable eco-efficient UHPC using normal mortar, recycled tire steel cords (RTSC), and recycled tire steel fibers (RTSF) [7]. Jabir studied the cracking impact resistance of UHPFRC [8]. Li studied the combined multi-point constraint multi-scale modeling strategy for ultra-high-performance steel fiber-reinforced concrete structures [9]. Pokorny studied the effect of elevated temperature on the bond strength of prestressing reinforcement in UHPC [10]. Xu studied the mechanism and microscopic morphology of steel-basalt mixed fiber HPCC [11]. Biswas studied the effects of steel fiber percentage and aspect ratios on the fresh and hardened properties of ultra-high-performance fiber-reinforced concrete [12]. Blasone studied ultra-high-performance fiber-reinforced concrete under impact [13]. Gayarre studied the use of waste from granite gang saws to manufacture ultra-high-performance concrete reinforced with steel fibers [14]. He studied the mechanical properties and damage evolution of UHPC reinforced with glass fibers and high-performance polypropylene fibers [15]. Huang studied the compressive behavior and modelling of CFRP-confined UHPC under cyclic loads [16]. Li studied the tensile behavior of hybrid fiber-reinforced UHPC [17]. Mala studied the mechanical and fracture parameters of ultra-high-performance fiber-reinforcement concrete cured via steam and water [18]. Niu studied the crack propagation behavior of UHPC reinforced with hybrid steel fibers under flexural loading [19]. Bermudez studied the shear behavior of UHPC beams with macro hooked-end steel fibers and PVA fibers [20]. Esmailzade studied the effect of the impurities of steel fibers extracted from shredded tires on the behavior of fiber-reinforced concrete [21]. Jang studied the effects of nano-SiO2 coating and induced corrosion of steel fiber on the interfacial bond and tensile properties of UHPC [22]. Niu studied the flexural behavior of UHPC with different steel fiber lengths [23]. Yu studied the effect of steel and polyoxymethylene fibers on the characteristics of UHPC [24]. Paulo investigated the performance improvement of fiber-reinforced cementitious composites after exposure to high temperatures [25]. Xiong studied the dispersion uniformity and performance improvement of steel fiber-reinforced lightweight aggregate concrete by vibrational mixing [26]. Ting Yang investigated the bond behavior between geopolymer-based UHPC and steel bars [27]. Yang studied the 3D-printing of ultra-high-performance fiber-reinforced concrete under triaxial confining loads [28]. Yu studied the dynamic behaviors assessment of steel fibers in fresh UHPC [29]. Zhou studied the torsional behavior of UHPC rectangular beams without steel reinforcements [30]. Ravichandran reviewed the influence of fibers on the fresh and hardened properties of UHPC [31]. Abdolpour reviewed the recycling of steel fibers and spent equilibrium catalysts in ultra-high-performance concrete [32]. Wang reviewed the fresh and rheological characteristics of fiber-reinforced concrete [33]. Chaopeng reviewed different testing methods and factors affecting the fracture properties of fiber-reinforced cementitious composites [34]. Daneshvar studied the thermal expansion and water loss of concrete under thermal action, regarding if it could cause cracks. These cracks may provide a way for direct heating of reinforcement, which may lead to higher thermal stress and greater cracking. More micro cracks and severe fire damage will reduce the strength of the plate under impact load [35]. Moradi developed a robust and time-saving method based on machine learning (ML) to predict the compressive strength of concrete containing binary supplementary cementitious material SCMs at various ages. It was found that SCM needs enough amorphous aluminosilicate to react with calcium hydroxide in the presence of water to form one or more hydration products: hydrated calcium silicate (C-S-H), hydrated calcium aluminate (C-A-H), and hydrated calcium aluminosilicate (C-A-S-H) [36].

The existing studies and reviews focused on the behavior of UHPC reinforced with micro straight steel fibers with a volume fraction of 1~3%. The corrugated and hooked BCSFs combine with UHPC to produce greater flexural strength due to closer mechanical engagement. Few studies on the influence of different types and amounts of high-performance brass-coated steel fibers on the flexural and compressive properties of UHPC have been reported. However, in our experiment, the flexural strength of mix UHPC with straight BCSFs was investigated. The BCSFs used to reinforce the UHPC flexural experiments were used to compare the mechanical properties of straight, hooked, and corrugated fibers under a 28-d curing period. It was found that the flexural strength and compressive strength of the BCSF-reinforced UHPC mix specimens are enhanced by increasing the content of BCSFs reinforcement from 1 wt.% to 6 wt.% with different shapes of straight, corrugated, and hooked fibers. The BCSFs easily cluster at high dosages during mixing, which directly limits the performance of concrete. We used vibrational mixing to overcome the defects of the ordinary mixing method. The cracking mechanism of different contents of the BCSFs–UHPC mixture and the BCSFs from bonding stage to pull-out stage was also discussed.

## 2. Materials and Method

### 2.1. Materials 

The UHPC matrix included 20-grain quartz sand, 40-grain quartz sand, 80-grain quartz sand, cement, silica fume, active superplasticizer, and defoamer. Mix proportions and notations are presented in Table 1. A polycarboxylate-based highly active superplasticizer was used to achieve the desired fluidity. This superplasticizer, in addition to fluidizing the fresh concrete, enables it to reach high strength in a short time. A water to cementitious materials (W/CM) ratio of 0.20 was adopted. In calculating the total water content in the mixture, the liquid phase in the superplasticizer and defoamer was considered. Different weight dosages of BCSFs (1 wt.%, 2 wt.%, 3 wt.%, 4 wt.%, 5 wt.%, and 6 wt.%) were selected as variables; 1–6 wt.% straight, corrugated, and hooked BCSFs were added to the UHPC, respectively, to investigate the effect of different content and types of BCSFs on the mechanical properties of UHPC. 

**Figure 1:**
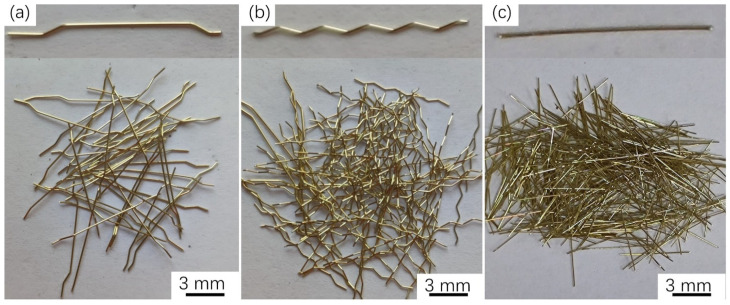
Types of the brass-coated steel fibers: (**a**) Hooked fibers, (**b**) Corrugated fiber, (**c**) Straight fiber.

Three types of BCSFs, including hooked fibers, corrugated fibers, and straight fibers with a length of 13 mm and diameter of 0.2 mm were selected for investigation, as illustrated in Figure 1. The properties of the three types of BCSFs (13/0.2) are summarized in Table 2.

### 2.2. Method

**Figure 2:**
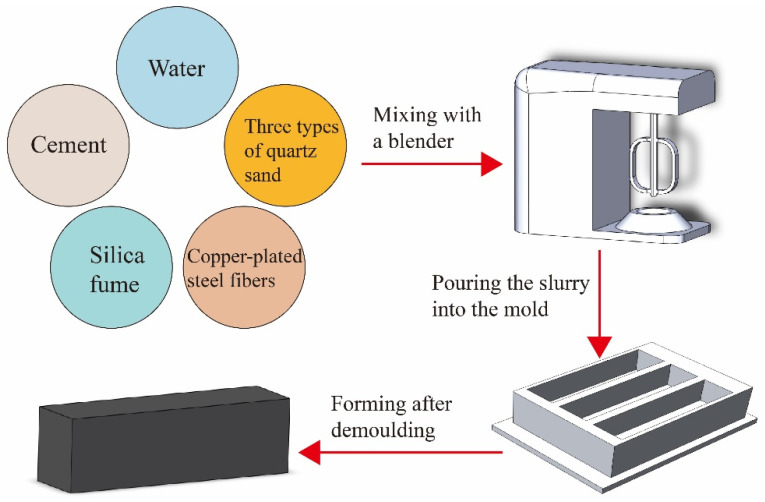
Preparation procedure of the brass-coated steel fiber-reinforced ultra-high strength concert.

The mixing procedure applied in this experiment is shown in Figure 2: 

(a) Binders (cement and silica fume) were placed in the mortar mixer and mixed dry for 1 min. (b) Water and superplasticizer additive were slowly added to the mixture, allowing it to mix for another 5 min. The rest of the dry components (powder and sand) were then added into the mixer and mixed for 2 min. (c) The BCSFs were added last and allowed to mix for another 3 min until uniformly distributed. Mixing speed was kept constant throughout the mixing process. 

The mixture was then cast into the mold in two steps.

**Figure 3:**
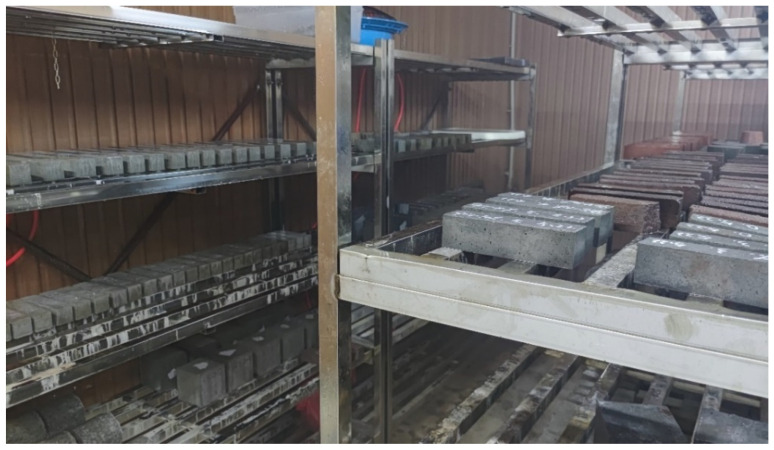
Specimens were cured for 7 days and 28 days in the standard curing room.

(a) First step. After the mixture slurry filled two-thirds of the mold, mechanical vibration of the mold was carried out to improve the compactness of the mixture in the mold. (b) Second step. After the mixture slurry completely filled the mold, mechanical vibration was continued for 3 min. (c) The specimens were demolded 24 h after casting and cured in the standard curing room until 7 days and 28 days, respectively, as illustrated in Figure 3. (d) The compressive strength and flexural strength of the BCSF-reinforced UHPC mix were tested after curing.

### 2.3. Characterization

**Figure 4:**
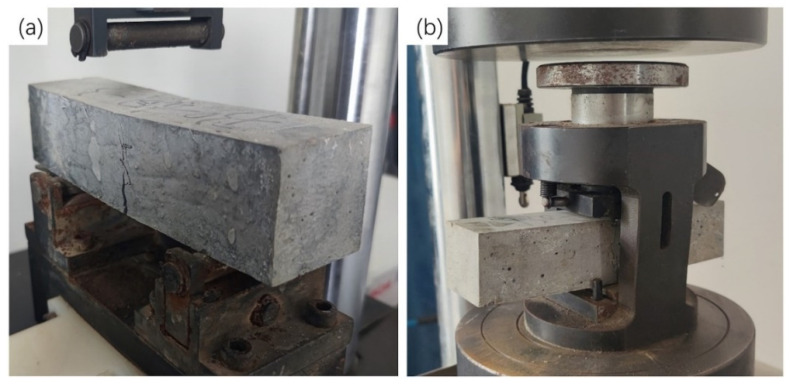
Mechanical properties testing of the BCSF-reinforced UHPC mix specimens: (**a**) flexural strength testing equipment; (**b**) Compressive strength testing equipment.

The flexural and compressive properties of BCSF-reinforced UHPC specimens cured for 7 days and 28 days were tested by a 3000 kN microcomputer-controlled electro-hydraulic servo pressure tester (YAW-3000D, Sansi Zhongheng, Shanghai, China), as shown in Figure 4. Three samples were used for each test.

**Figure 5:**
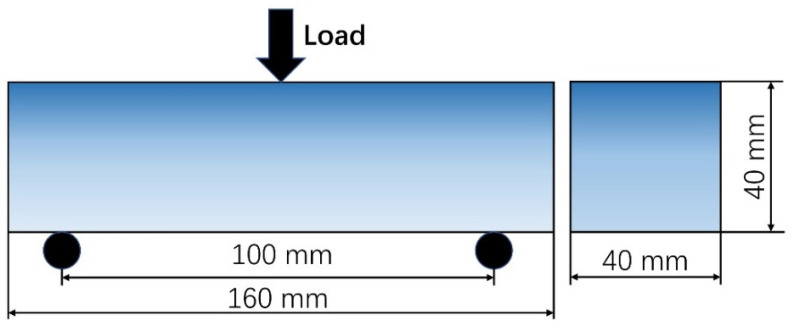
Technical drawings of the three-point bending test.

Flexural behavior of UHPC was assessed by performing the three-point bending test on specimens of 40 mm × 40 mm × 160 mm, with a span of 100 mm, as shown in Figure 5. 

The loading rate was taken as 50 N/s in the flexural tests, and the loading rate was taken as 2400 N/s in the compression tests. 

The variability of the results and the statistics are derived from three replicate formulae for each specimen [10].

**Figure 6:**
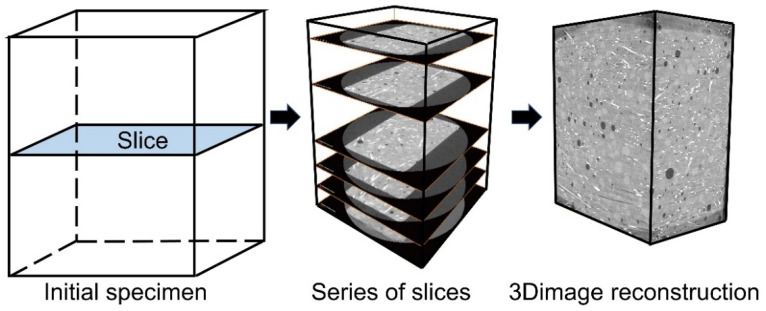
3D image reconstruction of the BCSF-reinforced UHPC mix specimens using X-ray computed tomography.

Field emission scanning electron microscopy (SEM, S-4800, Japan) and energy dispersive X-ray spectrometry (EDS, Oberkochen, Germany) were used for microscopic morphology, crack extension of the BCSF–matrix interface observation, and elemental distribution analysis. X-ray computed tomography (X-CT, Xradia 510 Versa, Germany) was used for the three-dimensional visualization of the matrix and/or BCSF–matrix interface, as shown in Figure 6. X-CT and Avizo 2020.1 software were used to measure the pore structure of the BCSF-reinforced mix UHPC. The penetration ability of X-rays is related to its current scanning voltage, the voltage used for CT is 150 kV, 67 μA, and the minimum pixel resolution is 32 μm. BCSFs and porosity were clearly distinguished by threshold segmentation of the X-CT images.

## 3. Results and Discussion

### 3.1. Effect of BCSF Content and Shapes on the Mechanical Properties of UHPC Mixtures

**Figure 7:**
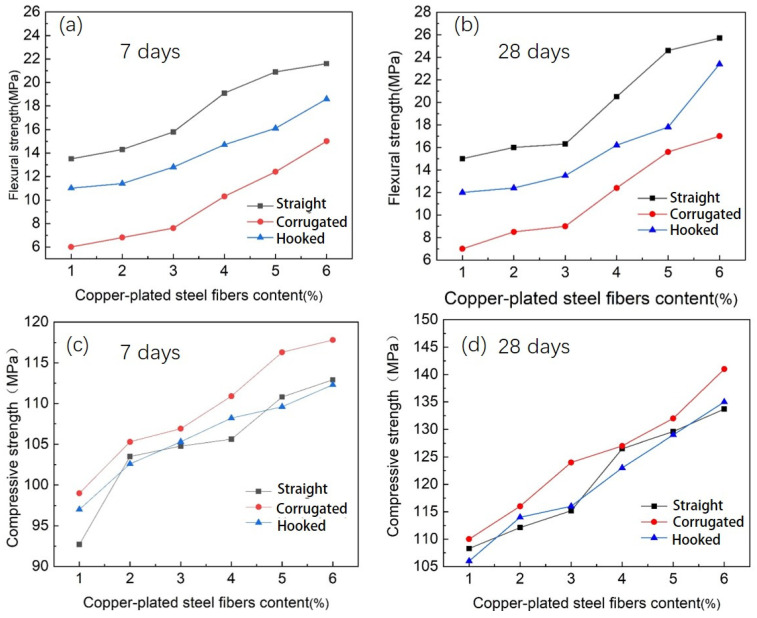
Flexural and compressive strength of three types of BCSF-reinforced UHPC with 1–6 wt.% cured at 7 days and 28 days: (**a**,**b**) 7 days; (**c**,**d**) 28 days; (**a**,**c**) flexural strength; (**b**,**d**) compressive strength.

Figure 7 shows the flexural strength and compressive strength of straight, hooked fibers and corrugated BCSF-reinforced UHPC specimens cured for 7 days and 28 days. As shown in Figure 7a, with 6 wt.% of BCSFs and cured at 7 days, the flexural strength of straight BCSF-reinforced UHPC specimens, corrugated BCSF-reinforced UHPC specimens, and hooked BCSF-reinforced UHPC specimens all reached the maximum values, which were 22.6 MPa, 18.9 MPa, and 15.1 MPa, respectively. With 6 wt.% of BCSFs cured for 28 days, the flexural strength of straight BCSF-reinforced UHPC specimens, corrugated BCSF-reinforced UHPC specimens, and hooked BCSF-reinforced UHPC specimens reached the maximum values, which are 26.2 MPa, 23.8 MPa, and 17.3 MPa, respectively, as shown in Figure 7b. Comparing Figure 7a,b, it can be seen that the incorporation of BCSFs obviously improved the mechanical strength of the mix specimens [17]. The carrying capacity of UHPC with straight BCSFs reached the highest flexural strength. The mechanical strength of the mix specimens increased with the increase in the BCSF content at 7 days and 28 days. The flexural strength of BCSF-reinforced UHPC specimens cured at 28 days increased slightly compared to that of UHPC specimens cured at 7 days. 

As shown in Figure 7c, with 6 wt.% of BCSFs and cured at 7 days, the compressive strength of the corrugated BCSF-reinforced UHPC specimen, the straight BCSF-reinforced UHPC specimen, and the hooked BCSF-reinforced UHPC specimen all reached the maximum values, which were 117.5 MPa, 112.6 MPa, and 112.2 MPa, respectively. With 6 wt.% of BCSFs and cured at 28 days, the compressive strength of the corrugated BCSF-reinforced UHPC specimen, hooked BCSF-reinforced UHPC specimen, and straight BCSF-reinforced UHPC specimen all reached the maximum values, which were 142.3 MPa, 135.2 MPa, and 134.6 MPa, respectively, as shown in Figure 7d. 

Comparing Figure 7c,d, it can be seen that the UHPC specimens cured at 28 days showed a significant increase in compressive strength compared to the UHPC specimens cured for 7 days. The UHPC specimens with corrugated BCSFs reached the highest compressive strength; however, UHPC with straight BCSFs reached the highest flexural strength. The flexural strength of the UHPC specimens reinforced with straight BCSF significantly exceeds those of the hooked and corrugated BCSF-reinforced UHPC specimens; however, the compressive performance of the UHPC specimens reinforced with straight BCSFs are almost equal to those of UHPC specimens reinforced with hooked and corrugated BCSFs. Moreover, the more BCSFs there are, the more pronounced the effect is. 

Therefore, it can be concluded that UHPC reinforced with straight BCSFs it is suitable for making BCSF-reinforced UHPC mixtures [26]. 

**Figure 8:**
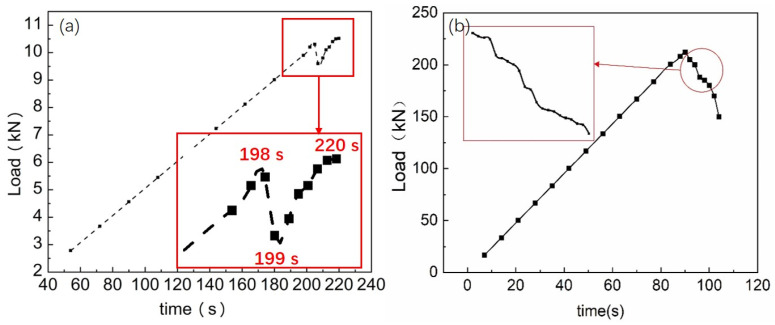
Physical properties of 6 wt.% straight BCSF-reinforced UHPC cured at 28 days during the load-bearing process: (**a**) flexural strength; (**b**) compressive strength.

The 6 wt.% straight BCSF-reinforced UHPC mixture specimens were selected to investigate the physical properties during the load-bearing process, as shown in Figure 8. The curve of the load-bearing process shows obvious zigzag patterns after the peak load, as shown in Figure 8a. The loaded force reached a peak maximum value of 10.5 kN at 198 s, the loaded force dropped steeply at 199 s due to the matrix being damaged, then the loaded force followed, increasing to 10.6 kN at 219 s. Finally, the specimen was fractured at 220 s. The curve indicates that the matrix of the test specimen did not fracture immediately when the cracking occurred at 198 s. Incorporating BCSFs not only hinders the development of micro-cracks at 198 s but also makes the specimen tougher and allows larger deformation before failure. When the matrix was destroyed, the load was basically carried by the BCSFs; even if the matrix is destroyed, the load can still continue to be slightly increased, and the UHPC mixture still had a certain load-bearing capacity at this time [22]. 

Therefore, straight BCSF-reinforced UHPC specimens could continue to sustain the load after the initial cracking.

In addition, a crisp “boom” sound, which could be heard clearly with the crack, was first observed after the peak load during the load-bearing process, which indicates that the BCSFs reinforcements were continuously pulled out from the matrix of the test specimen. This is consistent with the results in the literature [11]. However, the curve of compressive strength dropped abruptly during the load-bearing process, as shown in Figure 8b. It was observed that the micro-cracks on the surface of the specimen gradually develop into multiple macroscopic visible horizontal long cracks. 

**Figure 9:**
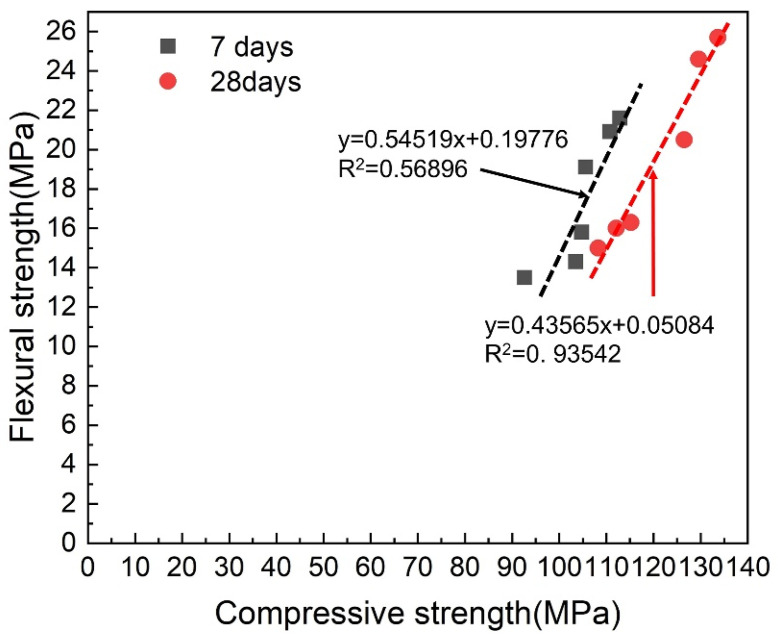
Correlation of compressive strength and flexural strength of the 6 wt.% straight BCSF-reinforced UHPC cured at 7 days and 28 days.

Correlation of compressive strength and flexural strength of the 6 wt.% straight BCSF-reinforced UHPC cured for 7 days and 28 days is investigated. It is observed that the flexural strength increases with the increase in compressive strength, as shown in Figure 9. Due to the fact that changes in the series are reasonable, a considerably high correlation coefficient (i.e., 0.93542) was obtained [4].

### 3.2. CT Observation of 1–6 wt.% Straight BCSF-Reinforced UHPC Mixtures 

**Figure 10:**
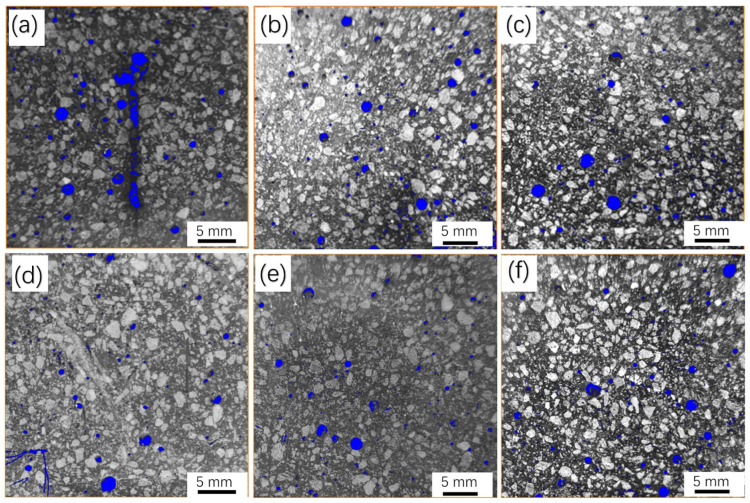
Macrostructure of straight BCSF-reinforced UHPC with varying content of 1–6 wt.% cured for 28 days: (**a**) 1%; (**b**) 2%; (**c**) 3%; (**d**) 4%; (**e**) 5%; (**f**) 6%.

Figure 10a–f show the macrostructure of straight BCSF-reinforced UHPC specimens with varying content of 1–6 wt.% cured for 28 days by CT observation, respectively. The BCSFs and pores in the UHPC mixture can be clearly distinguished by threshold segmentation image processing. The blue circles represent the pores of the specimens. It can be observed that the porosity of the specimens decreases gradually with the increase in BCSF content. This non-uniform distribution of pores may lead to variations in strength at different locations in the specimens. The mechanical strength of the UHPC mixtures was reduced by the introduction of more air bubbles and an uneven distribution of BCSFs [31]. 

Table 3 lists the porosities of the specimens shown in Figure 10a–f by Avizo software analysis. The porosity decreases from 18.4% to 15.2% when the straight BCSF content increases from 1 wt.% to 2 wt.%. However, the porosity decreases from 9.6% to 8.3% when the straight BCSF content increases from 5 wt.% to 6 wt.%. It is revealed that the change in porosity gradually decreases as the content increases. This may be due to the BCSFs destroying some of the large air bubbles within the slurry mixture during the mixing and casting process, thus leading to reduced content of large air bubbles in the UHPC mixture. The 6 wt.% straight BCSF-reinforced UHPC specimen contains the minimum porosity and the minimum average pore diameter, resulted in a denser microstructure and better bond properties. 

Therefore, it obtained the highest flexural and compressive strength, which is consistent with the tested data in Figure 7.

### 3.3. CT Observation of 6 wt.% Straight BCSF-Reinforced UHPC Mixture

**Figure 11:**
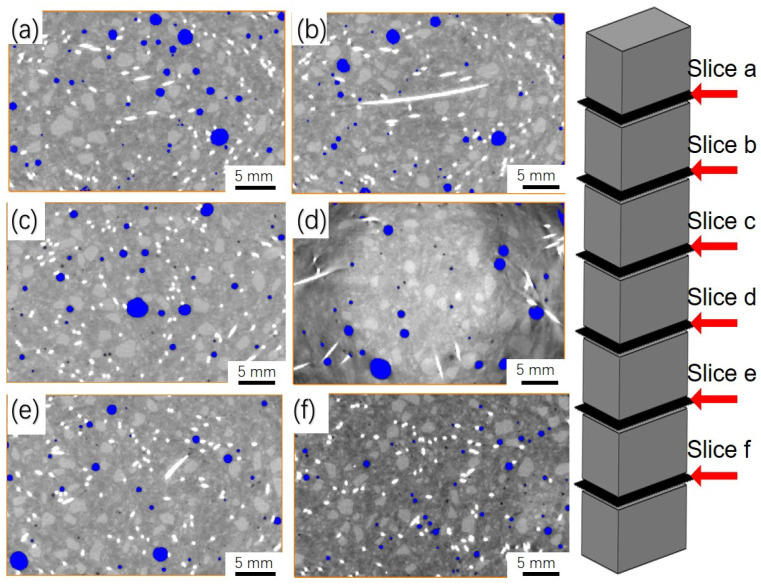
From the top to the bottom, the reconstructed 3D image of 6 wt.% straight BCSF-reinforced UHPC at 28 days: (**a**) slice a; (**b**) slice b; (**c**) slice c; (**d**) slice d; (**e**) slice e; (**f**) slice f.

The reconstructed 3D images of 6 wt.% straight BCSF-reinforced UHPC at 28 days by CT observation are divided into six equal parts from the top to the bottom, as shown in Figure 11. The BCSFs and pores in the UHPC mixture can be clearly distinguished by threshold segmentation image processing. The circular apertures in Figure 11 are the pores, while the short silver-white rods are the BCSFs.

Table 4 lists the porosities of the specimens shown in Figure 11a–f by Avizo software analysis. It can be seen that the porosity decreased from 12.3% to 8.2%, and the average pore diameter decreased from 0.5 mm to less than 0.1 mm from the top (slice a) to the bottom (slice f). The spacing between large pores is close at the upper parts, as shown in Figure 11a,b. The density of BCSFs gradually increases and the number of large pores significantly reduced from the upper parts to the lower parts, as shown in Figure 11c,d. During the process of pouring slurry into the mold, mixed slurry introduced air bubbles into the slurry, even in the experiment using mechanical vibration to exclude the air bubbles from the slurry mixture. This uneven distribution of BCSFs and pores will lead to a local reduction in the mechanical properties of the entire mix. This corresponds well with the previous bond properties to BCSFs in Figure 10.

Comparing Figure 11a–f, it can be seen that an appropriate content of BCSFs improves the stability of the slurry mixture. The problem of the uneven distribution of BCSFs and pores in the UHPC mixture can effectively be solved by adding 6 wt.% BCSFs.

### 3.4. Effect of Straight BCSF Contents on Crack Patterns of UHPC Mixture

**Figure 12:**
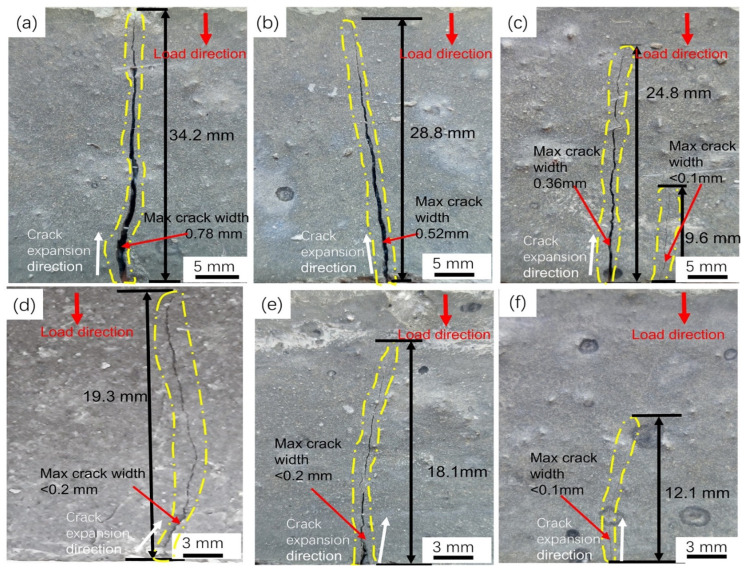
Crack patterns of straight BCSF-reinforced UHPC specimens with varying content of 1–6 wt.% at 28 days after flexural strength test: (**a**) 1%; (**b**) 2%; (**c**) 3%; (**d**) 4%; (**e**) 5%; (**f**) 6%.

The crack patterns of straight BCSF-reinforced UHPC specimens with varying content of 1–6 wt.% at 28 days after flexural strength test is shown in Figure 12. The disordered distribution of BCSFs can span both sides of the crack, as shown in Figure 12a–f. With BCSFs incorporated, the UHPC mixture does not break directly after flexural testing, but it shows a certain degree of flexural strength, providing a ductile damage mode for the UHPC mixture [3,28]. With the BCSF content increased from 1 wt.% to 6 wt.%, the max crack length of the UHPC mixture is reduced from 34.2 mm to 12.1 mm, and average crack width length decreased from 0.78 mm to less than 0.1 mm, as shown in Table 5. 

The UHPC mixture specimen with 1 wt.% BCSFs broke and cracked to form macro-cracks after reaching the peak stress, showing brittle damage, as shown in Figure 12a,b. During crack extension, part of the energy in the UHPC mixture specimen flowing into the crack tip region is converted into the work of the fracture process dissipated during the fracture separation process [3,5,31]. 

However, with BCSFs increasing to 3 wt.% and 4 wt.%, UHPC mixture specimens exhibit noticeable differences, as shown in Figure 12c,d. Multiple crack zones are clearly visible for the UHPC mixture specimen with 3 wt.% BCSFs in Figure 12c. Part of the energy in the UHPC mixture specimen flowing into the crack tip region is converted into the work of the peripheral plastic dissipation, excited by the high stress in the fracture tip region during the separation process. The micro-cracks develop into macroscopically visible cracks with the appearance of multiple vertical micro-cracks with increased loading [34]. This means the specimen can absorb more energy and have better toughness than that shown by the other specimens.

The diverse and complex strains that appear at the crack tips are clearly visible for the UHPC mixture specimen with 3 wt.% BCSFs, as shown in Figure 12d. The increase in BCSFs leads to the increase in energy required for debonding, making it necessary to generate other cracks to dissipate the remaining energy. The more curved the crack surface is, the greater the consumption of fracture surface energy.

With BCSFs increasing to 5 wt.% and 6 wt.%, the UHPC mixture specimens exhibited progressive BCSFs pullout and similar load-deflection behavior, as shown in Figure 12e,f [16,31]. The increased amount of BCSFs enhanced the interaction of fibers with each other, resulting in an increase in mechanical characteristics. During crack extension, part of the energy in the UHPC mixture specimen with the 6 wt.% BCSF content flows into the crack tip region, converted into the work dissipated during the bridging process, as shown in Figure 12e [9]. After crack initiation, the BCSF bridging effect is activated and bears the external loading, as shown in Figure 12f [17]. 

Combined with the results of the three-point flexural test, it can be concluded that BCSFs can control the opening of diagonal cracks and enhance aggregate interlocking [5]. When the concrete specimen is loaded, the micro cracks converge, expand, and penetrate the matrix, eventually causing overall cracking damage of the specimen. After the incorporation of BCSFs into UHPC, the expansion of cracks in the UHPC will be hindered by the expansion from the BCSFs; the size of the hindering force is related to the amount of BCSFs incorporated [32], and with the matrix cracking, BCSFs can still play a bridging role, making it a larger slow crack expansion before the damage [19]. The displacement of the open end of the crack decreases with the increase in BCSFs. The cracks become more irregular, and the fracture energy and the fracture toughness of the UHPC mixture increases with the increase in the BCSF content. 

**Figure 13:**
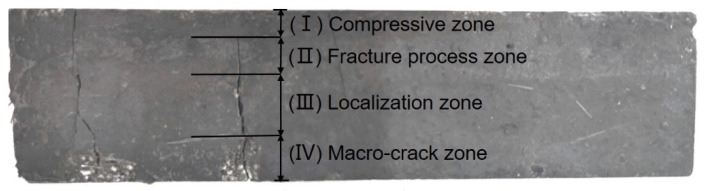
Straight BCSF-reinforced UHPC of 6wt.% content cured for 28 days after bending test flexural strength test.

Figure 13 demonstrates that the area around the crack tip may be divided into four zones based on the strain distribution [19]: (I) a compressive zone near the loading point; (II) fracture process zone where the fibers and UHPC matrix are de-bonded (the full debonding displacement between the fibers and the matrix is 0.022 mm); (III) localization zone where the micro-crack is located; and (IV) a macro-crack zone where the crack is visible to the naked eye [26]. In the compressive zone, the maximum compressive strain emerged at the top of the beam and decreased along the longitudinal axis. When the compressive strain value reached zero, the fracture process zone appeared and the maximum crack width was 0.036 mm. The localization zone ended, and the maximum strain at the crack tip could be obtained when the crack width was 0.06 mm [19,23].

**Figure 14:**
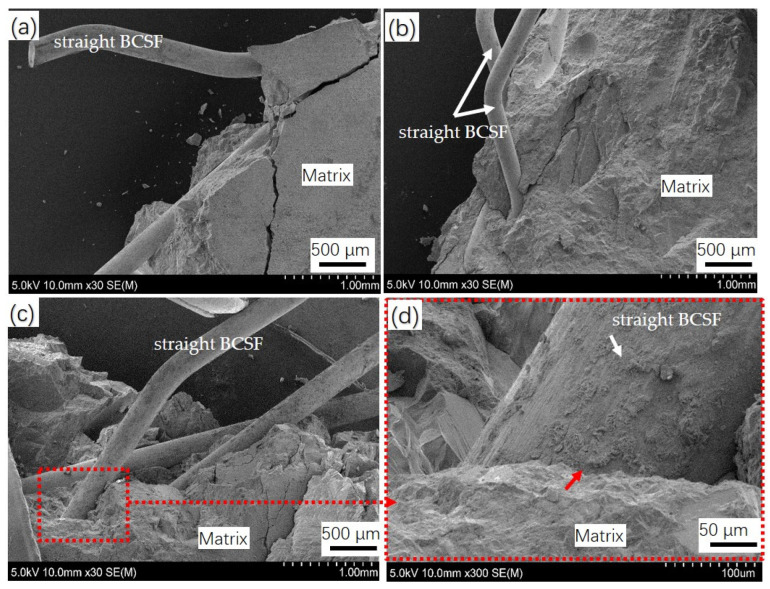
SEM imagines of crack growth of straight BCSF-reinforced UHPC of different content cured for 28 days after compressor strength test: (**a**) 2 wt.%; (**b**) 4 wt.%; (**c**) 6 wt.%; (**d**) magnified image in the red box area of (**c**).

Figure 14 shows the SEM images of the crack growth of straight BCSF-reinforced UHPC with a content of 2 wt.%, 4 wt.%, and 6 wt.%, cured for 28 days after the compressor strength test. It can be seen that the incorporation of BCSFs considerably influenced the failure patterns of the UHPC. The ductility of the BCSF-reinforced UHPC is not obvious; multiple macro-cracks appeared on the UHPC mixture specimen with 2 wt.% BCSFs, and the fracture was smooth, as shown in Figure 14a.

By adding BCSFs up to 4 wt.%, the number of macro-cracks was effectively reduced, and ribbon-like failure fragments were generated, as shown in Figure 14b. More energy was needed to overcome the bridging effect; thus, the inclusion of the UHPC mixture specimen with 4 wt.% BCSFs arrested the crack propagation [1]. 

By adding BCSFs up to 6 wt.%, the BCSFs showed a larger peak strain of the specimen and slower macro-crack evolution after cracking [31]. Because the interface between the BCSFs and UHPC matrix is very dense, more energy is needed to separate the BCSF and UHPC matrix with the increase in BCSF content. Near the surface plane of the specimen, the BCSFs located in the crack path and not perpendicular to the cracking direction would be subjected to shear during the splitting process, resulting in localized spalling of the matrix and the formation of ribbon fragments, as seen in Figure 14c,d [5]. 

**Figure 15:**
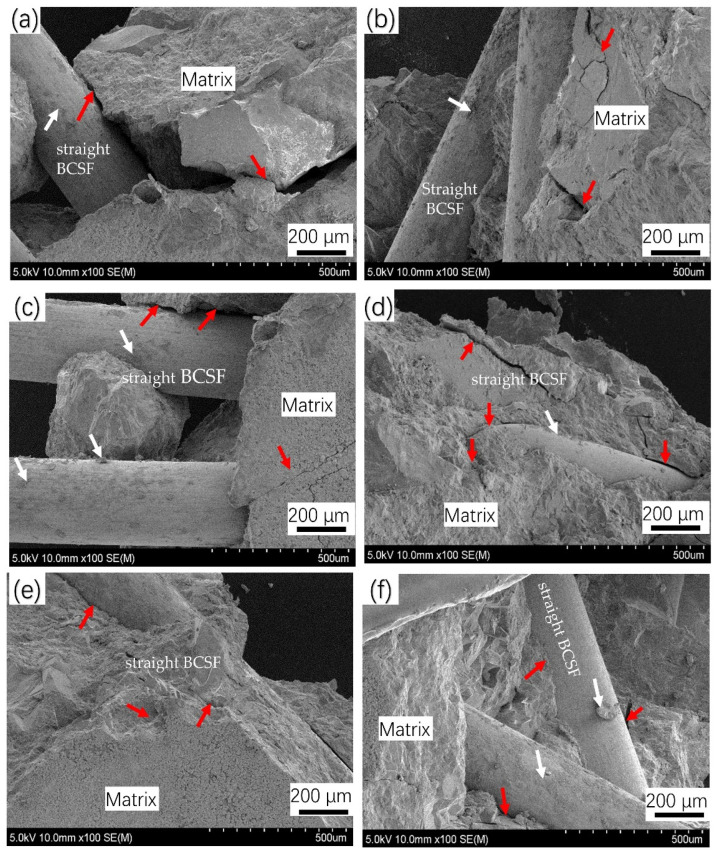
SEM imagines of the crack growth of straight BCSF-reinforced UHPC of different content cured for 28 days after flexural strength test: (**a**) 1%; (**b**) 2%; (**c**) 3%; (**d**) 4%; (**e**) 5%; (**f**) 6%.

Figure 15 shows SEM imagines of the crack growth of straight BCSF-reinforced UHPC with varying content of 1–6 wt.% at 28 days after flexural strength test. Different from the compressor strength test, in the bending test, more BCSFs produce obvious pull-out damage. Based on the SEM imagines, the pullout behavior of the inclined BCSFs distributed on the fracture surface can be divided into the perfect bonding stage, partial debonding stage, and fiber pullout stage. The perfect bonding stage is the elastic stage. The load before initial cracking increases linearly, and BCSFs bear force cooperating with the UHPC matrix because of its high elastic modulus. In the partial debonding stage and fiber pullout stage, the load declines to brittle failure rapidly after initial cracking, and it is then difficult for small-sized BCSFs to bridge macro cracks, causing BCSFs to be continuously and rapidly pulled out.

Multiple macro-crack and BCSF de-bonding from the matrix was observed on the UHPC mixture specimen with 1 wt.% and 2 wt.%, as seen in Figure 15a,b. The BCSFs begin to de-bond from the matrix when the pullout load is between critical and maximum loads. It was seen that the debonding zone extends to the end of the embedded BCSFs [23]. In Figure 15c,d, the BCSFs have a smooth surface and a small number of hydration products on the surface, which meant that the interfacial bonding strength between the matrix and the BCSFs was weak [17]. The weaker bond strength and the higher tensile strength of BCSFs made it easier for the BCSFs to pull out rather than rupture. However, with BCSFs increasing to 5 wt.% and 6 wt.%, the UHPC mixture specimens exhibit noticeable differences compared with the specimens in Figure 15c,d. 

A key factor of the reinforcing effect of the BCSFs is the average spacing between BCSFs. The smaller the average spacing, the stronger the binding force of the BCSFs against the further expansion of cracks and the higher the strength of BCSFs concrete. As shown in Figure 15e,f, the BCSFs distributed within the matrix play a certain role in the further development of the crack. They maintain the good integrity of the concrete matrix and strengthen the mechanical bite force of the matrix on both sides of the crack. The BCSF surface has obvious debonding, which means that during the debonding process, the fiber part elastically bonded to the matrix becomes shorter, and the shear force is mainly controlled by friction. In this manner, the internal stress of the material becomes well transmitted and dissipated.

In Figure 15e,f, it can be seen that perfect crack bridging effect appeared on the UHPC mixture specimen with 5 wt.% and 6 wt.%. BCSFs that cannot be easily de-bonded from a matrix with high compactness and strength increased the flexural strength, resulting in the increased crack bridging. 

In this experiment, vibrational mixing was used to overcome the problem of clustering at high dosages during mixing. It was revealed that the bond strength between the BCSFs and UHPC matrix was enhanced by using vibrational mixing. BCSFs can effectively prevent the propagation of macro-cracks and improve the ductility of materials until most of the BCSFs were pulled out. In the early stage of failure, the increased amount of BCSFs can effectively play a bridging role and delay the further development of micro-cracks. This is consistent with findings available in the literature on the effect of manufactured steel fiber length on the flexural strength of UHPC [25,26].

### 3.5. BCSFs Surface 

**Figure 16:**
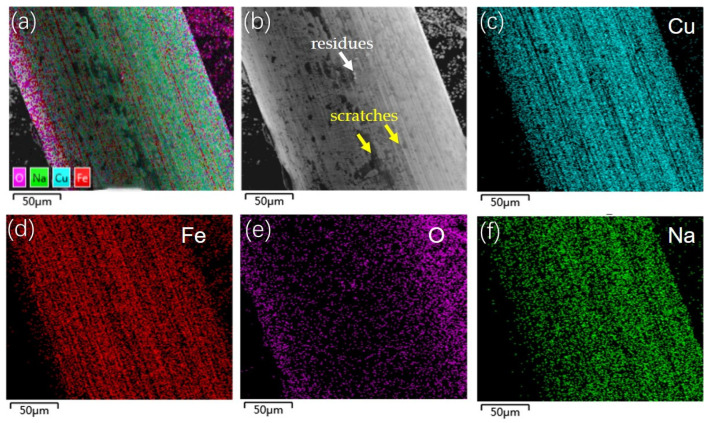
SEM and EDS images of straight BCSFs: (**a**) EDS imagine; (**b**) SEM imagine; (**c**) Cu; (**d**) Fe; (**e**) O; (**f**) Na.

The BCSF surfaces were characterized using SEM analyze to investigate the surface of the BCSFs after the bending and compressive tests, as shown in Figure 16. A low roughness film is coated on the surface of the BCSFs, as shown in Figure 16a,b. Some scratches (yellow arrow marks) aligned along the axial direction length of the BCSF, which resulted from the contact of the BCSFs surface with the UHPC matrix during the bending and compressive test, as shown in Figure 16b. Residues (white arrow mark in Figure 16b) were observed on the BCSFs surface, proving that the UHPC matrix adhered to the BCSF surface. The brass film promoted a physical–chemical bond and frictional resistance between the BCSFs and the UHPC matrix owing to the increase in surface roughness, as shown in Figure 16b by EDS analyze. Fe element (Figure 16d), element O (Figure 16e), and Na element (Figure 16f) were also found by EDS analysis, suggesting that the O and Na elements in the matrix diffused to the surface of the BCSFs through the BCSFs–matrix interface because the coated brass film favors adhesion with the HPC matrix [23]. The increased additional adhesion indicated a significant BCSFs–matrix adhesion, which contributes to increasing the flexural and compressive strength of the UHPC mixture.

### 3.6. BCSFs–Matrix Interface 

**Figure 17:**
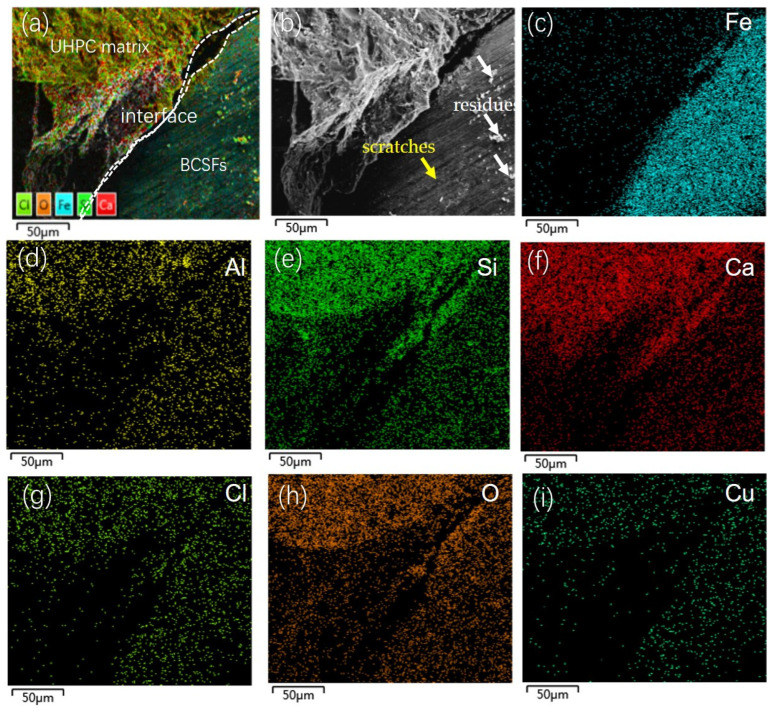
SEM and EDS images of interface of 6wt.% straight BCSF-reinforced UHPC specimens at 28 days: (**a**) EDS imagine; (**b**) SEM imagine; (**c**) Fe; (**d**) Al; (**e**) Si; (**f**) Ca; (**g**) Cl; (**h**) O; (**i**) Cu.

Figure 13 shows SEM images of the BCSFs–matrix interface of 6 wt.% straight BCSF-reinforced UHPC specimens at 28 days. The BCSFs–matrix interface (white dotted line mark) is integrity, and the brighter white area represents the matrix, which exhibited a homogeneous constitution with grains of varying sizes and shapes randomly distributed, as shown in Figure 17a. The embedded BCSFs are represented by darker areas, as shown in Figure 17b. The brass film of the BCSF surface presented some scratches in Figure 12b (yellow arrow mark) along the axial direction, and residues (white arrow mark) were observed on the BCSFs surface in Figure 12b. Some scratches on the brass film surface can improve adhesion of the matrix surface in contact with the BCSFs, resulting in optimization of the BCSFs–matrix interface. Less unhydrated cement particles and smaller porous areas were observed around the embedded BCSFs, which leads to a denser microstructure. The BCSFs–matrix interface is favored by densification of the UHPC matrix and surface alteration of the BCSFs [23]. Fe (Figure 17c), Al (Figure 17d), Si (Figure 17e), Ca (Figure 17f), Cl (Figure 17g), O (Figure 17h), and Cu (Figure 17i) were also found by EDS analysis, suggesting that these elements in the matrix diffused to the surface of the BCSFs through the BCSFs–matrix interface because the specimen has better bonding properties and less micro-cracking of the BCSFs–matrix interface.

Therefore, BCSFs are immersed in the cementitious matrix to maintain high flexural resistance. At higher strain rates, the debonding of BCSFs on the initial crack path was insufficient to consume enough energy, making it necessary to generate other cracks to dissipate the remaining energy. 

The micro cracks converged, expanded, and penetrated the matrix, eventually causing an overall cracking damage of the specimen during the UHPC mixture specimen loading process. The BCSFs mainly reduced the scale and quantity of the cracks, mitigated the degree of stress concentration at the tip of crack propagation, and inhibited the occurrence and expansion of cracks. 

In this study, the flexural strength and compressive strength of BCSF-reinforced UHPC mixture specimens were enhanced, along with increasing the content of BCSF reinforcement with different shapes. We have not discussed the effect of BCSF content on the mechanical properties of UHPC when the content of BCSF exceeds 6%. However, increasing the content of BCSF may reduce the strength of UHPC. Considering the diversity of the mix and the complexity of influencing factors, we will investigate the internal relationship between the fiber content and macro strength in future research. In addition, BCSF pull-out tests will be used to determine the interface parameters, such as the bonding and friction coefficients.

### 3.7. Crack Expansion of Straight BCSF-Reinforced UHPC

**Figure 18:**
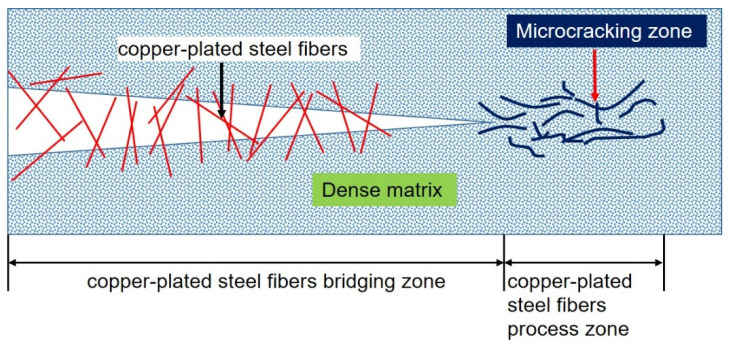
Schematic description of the relationship between BCSFs bridging and matrix softening.

The relationship between BCSF bridging and matrix softening is shown in Figure 18. The crack tip is divided into two different zones. The first zone is the BCSFs bridging zone. In this zone, the BCSFs were partially deboned from the matrix (i.e., part of it is deboned, while the other part is fully bonded), and the interfacial bond stress increases linearly as the deboned area extends to the embedded end of the BCSFs. The majority of the external load is carried by fiber bridging. The second zone is the BCSFs process zone, where the micro-crack appears [30]. BCSFs bridging and matrix softening are the main factors affecting the flexural behavior of UHPC mixtures. The BCSFs process zone also affects flexural behavior by hindering the propagation of cracks and extending the area of stress concentrate [19]. 

## 4. Conclusions

1. The flexural strength and compressive strength of BCSF-reinforced UHPC mixture specimens are enhanced by increasing the weight fraction of embedded BCSF reinforcement with different shapes (straight, corrugated, and hooked).
2. BCSFs play a major role in the adhesive properties and stress transfer of the interface between reinforced BCSFs and the UHPC matrix. The flexural strength of UHPC with straight BCSFs is higher than those with corrugated and hooked fibers. However, the compressive strength of UHPC with corrugated BCSFs is higher than those with straight and hooked BCSFs. The flexural strength of UHPC mixture with 6 wt.% straight BCSFs at 28 days reaches the maximum value of 26.2 MPa, and the compressive strength of UHPC with 6 wt.% corrugated BCSFs at 28 days reaches the maximum value of 142.3 MPa.
3. With the increase in straight BCSF content from 1 wt.% to 6 wt.%, the porosity in the UHPC mixture reduces gradually from 18.4% to 8.3%.
4. The crack propagation resistance of mix UHPC with straight BCSFs is slightly improved compared with those with corrugated and hooked BCSFs. With the increase in straight BCSF content from 1 wt.% to 6 wt.%, the crack length reduced from 34.2 mm to 12.1 mm, and average crack width reduced from 0.78 mm to less than 0.1 mm.
5. The brass film promoted a physical–chemical bond and frictional resistance between the BCSFs and UHPC matrix owing to the increase in surface roughness, which contributes to increasing the flexural and compressive strength of the UHPC mixture.

## Figures and Tables

**Table 1:** Weight ratios of the material ingredients for the ultra-high-performance concrete mixture.

Number	Brass-Coated Steel Fibers Content /wt.%	20-Grain Quartz Sand /g	40-Grain Quartz Sand /g	80-Grain Quartz Sand /g	Cement /g	Silica Fume /g	Active Superplasticizer /g	Defoamer /g
1	1	330	330	330	880	220	6.4	0.32
2	2	330	330	330	880	220	6.4	0.32
3	3	330	330	330	880	220	6.4	0.32
4	4	330	330	330	880	220	6.4	0.32
5	5	330	330	330	880	220	6.4	0.32
6	6	330	330	330	880	220	6.4	0.32

**Table 2:** Properties of the three selected brass-coated steel fibers.

Type	Length (mm)	Diameter (mm)	Aspect Ratio	Density	Tensile Strength (MPa)	Modulus of Elasticity (GPa)
Corrugated	13	0.2	65	7.8	>2000	200
hooked	13	0.2	65	7.8	>2000	200
Straight	13	0.2	65	7.8	>2000	200

**Table 3:** Porosity of straight BCSF-reinforced UHPC mixtures with 1–6 wt.% cured for 28 days.

Content of BCSFs	1 wt.%	2 wt.%	3 wt.%	4 wt.%	5 wt.%	6 wt.%
Porosity of UHPC mixture	18.4%	15.2%	13.6%	11.7%	9.6%	8.3%
Average pore diameter	0.8	0.7	0.5	0.4	0.3	<0.1

**Table 4:** Porosity of 6 wt.% straight BCSF-reinforced UHPC mixtures from the top to the bottom cured for 28 days.

Slice Location	a	b	c	d	e	f
Porosity of UHPC mixture	12.3%	11.2%	11.6%	10.4%	9.6%	8.2%
Average pore diameter, mm	0.5	0.4	0.3	0.2	<0.1	<0.1

**Table 5:** Crack patterns of straight BCSF-reinforced UHPC specimens with 1–6 wt.% at 28 days after flexural strength test.

Content of BCSFs	1 wt.%	2 wt.%	3 wt.%	4 wt.%	5 wt.%	6 wt.%
Max crack length, mm	34.2	28.8	24.8	19.3	18.1	12.1
Average crack width, mm	0.78	0.52	0.36	<0.2	<0.2	<0.1

## Data Availability

Data sharing is not applicable for this article.
